# *De novo* Genome Assembly of the Raccoon Dog (*Nyctereutes procyonoides*)

**DOI:** 10.3389/fgene.2021.658256

**Published:** 2021-04-29

**Authors:** Luis J. Chueca, Judith Kochmann, Tilman Schell, Carola Greve, Axel Janke, Markus Pfenninger, Sven Klimpel

**Affiliations:** ^1^LOEWE-Centre for Translational Biodiversity Genomics (LOEWE-TBG), Senckenberg Nature Research Society, Frankfurt am Main, Germany; ^2^Department of Zoology and Animal Cell Biology, University of the Basque Country (UPV-EHU), Vitoria-Gasteiz, Spain; ^3^Senckenberg Biodiversity and Climate Research Centre (SBiK-F), Frankfurt am Main, Germany; ^4^Institute for Ecology, Evolution and Diversity, Goethe University, Frankfurt am Main, Germany; ^5^Institute of Organismic and Molecular Evolution, Faculty of Biology, Johannes Gutenberg University, Mainz, Germany

**Keywords:** genome assembly and annotation, SARS-CoV-2, Carnivora, raccoon dog (*Nyctereutes procyonoides*), B chromosome

## Introduction

**Figure 1:**
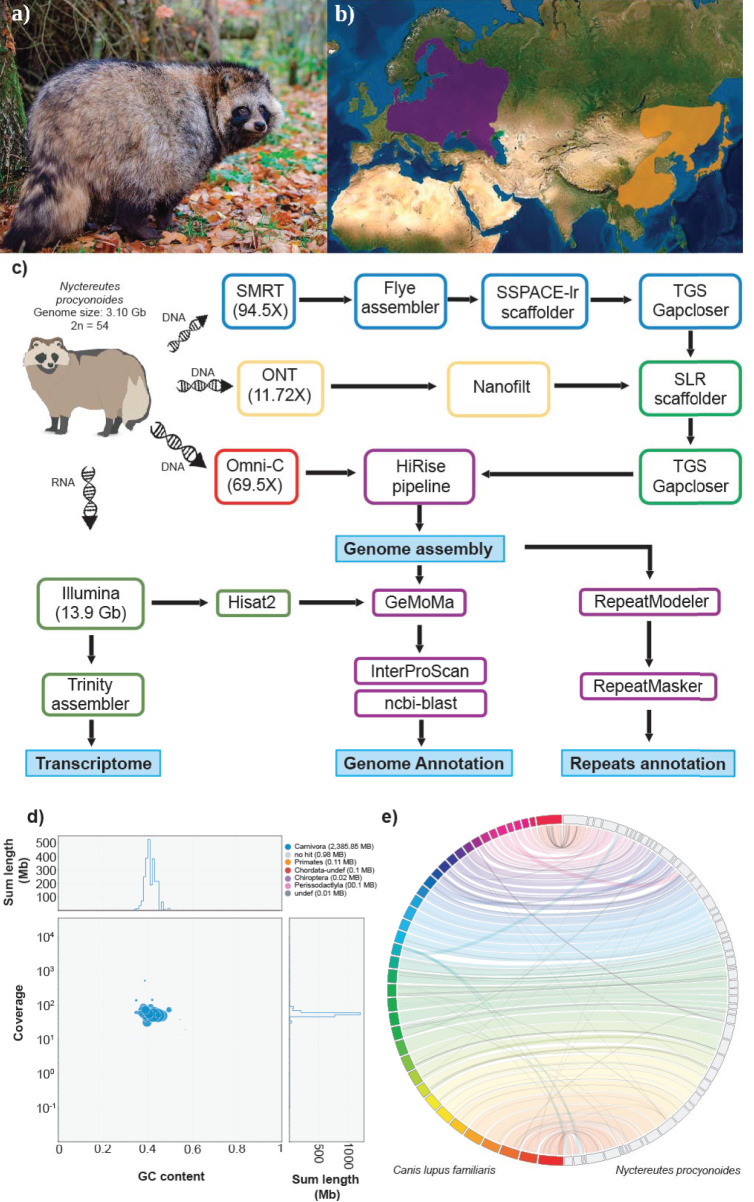
**(a)** Picture of an adult specimen of raccoon dog (*Nyctereutes procyonoides*), copyright © Dorian D. Dörge. **(b)** Native (orange) and introduced (purple) distributions ranges of raccoon dog (source IUCN red list). **(c)** Workflow of genome assembly and annotation followed in this study. **(d)** Blob plot showing read depth of coverage, GC content and size of each scaffold. Size of the blobs correspond to size of the scaffold and color corresponds to taxonomic assignment of BLAST (blue = Carnivora). **(e)** Whole genome synteny, obtained with Jupiterplot, between the *Canis lupus familiaris* chromosome-level assembly (on the left) and the raccoon dog genome assembly obtained in this study (on the right). The lines indicate aligned regions between the two assemblies.

The raccoon dog, *Nyctereutes procyonoides* (NCBI Taxonomy ID: 34880, Figure 1a) belongs to the family Canidae, with foxes (genus *Vulpes*) being their closest relatives (Lindblad-Toh et al., 2005; Sun et al., 2019). Its original distribution in East Asia ranges from south-eastern Siberia to northern Vietnam and the Japanese islands. In the early 20th century, the raccoon dog was introduced into Western Russia for fur breeding and hunting purposes, which led to its widespread establishment in many European countries, Figure 1b. Together with the raccoon (*Procyon lotor*), it is now listed in Europe as an invasive species of Union concern (Regulation (EU) No. 1143/2014) and member states are required to control pathways of introductions and manage established populations.

The raccoon dog is a host and vector for a variety of pathogens, including rabies and canine distemper virus. Whether, it is involved in the transmission of coronaviruses to humans is inconclusive (Guan, 2003; Chan and Chan, 2013), but experimental studies have demonstrated that raccoon dogs are susceptible to SARS-CoV-2 infection and its transmission to contact animals (Freuling et al., 2020). However, a recent study using predictions by sequence alignment suggests that the mammalian ACE2 receptor of *N. procyonoides* binds less effectively to the S-protein of SARS-CoV and SARS-CoV-2 than those of other species like cows and rodents (Luan et al., 2020a,b).

Several subpopulations have been recognized in their current range of distribution in Europe and East Asia based on mtDNA (Kim et al., 2013; Paulauskas et al., 2016), microsatellite (Drygala et al., 2016; Hong et al., 2018), and SNP markers (Nørgaard et al., 2017). Interestingly, continental populations from Asia and Europe seem to have a higher number of chromosomes (2*n* = 54) than those from Japanese islands (2*n* = 38) (Wada and Imai, 1991; Wada et al., 1991; Nie et al., 2003). Moreover, the raccoon dog is also known to be one of the few Carnivora species which presents B chromosomes (Bs) in its karyotype (Duke Becker et al., 2011; Makunin et al., 2018). Several mitochondrial genome sequences of wild and bred raccoon dogs are known (Sun et al., 2019), however, a complete nuclear genome is not still available. Apart from its potential role as disease vector, *N. procyonoides* is of interest because it is the only extant species in the genus *Nyctereutes* and the only canid known to hibernate.

Here, a first draft genome of a raccoon dog sampled in Germany is presented, which will provide a basis for deeper understanding of its phylogenetic relationships, the evolution and function of B chromosomes in mammals, give insights in the evolution of hibernation, provide markers for future studies on invasive population structures in Europe and serve as a resource for studying gene-disease associations.

## Materials and Methods

### Sample Collection, Library Construction, Sequencing

One adult female individual of raccoon dog, *Nyctereutes procyonoides* (Figure 1a), was bagged in February 2020 in Germany (52°06′51.2″N 12°03′03.6″E) according to hunting regulations. Blood samples as well as various types of tissue were immediately stored on dry ice or in RNAlater and kept at −80°C until further processing (Figure 1c).

A SMRTbell library was constructed following the instructions of the SMRTbell Express Prep kit v2.0 with Low DNA Input Protocol (Pacific Biosciences, Menlo Park, CA). Blood (5 ml) was used for high molecular weight DNA extraction using Genomic-tip 100/G (QIAGEN) according to the manufacturers' instructions. One SMRT cell sequencing run was performed in CLR mode on the Sequel System II with the Sequel II Sequencing Kit 2.0. For chromosome-level genome information, genomic DNA was isolated from ear tissue (62 mg) following the OMNI-C Proximity Ligation Assay (Version 1.1) with some modifications. The library was sequenced on the NovaSeq 6000 platform using a 150 paired-end sequencing strategy at Novogene (UK). The fragment size distribution and concentration of each of the final libraries was assessed using the TapeStation (Agilent Technologies) and the Qubit Fluorometer and Qubit dsDNA HS reagents Assay kit (Thermo Fisher Scientific, Waltham, MA), respectively. For more information on the different protocols see Supplementary Information.

To obtain Oxford Nanopore Technologies (ONT) long reads, we ran three flow cells on a MinION portable sequencer (FLO-MIN106). Total genomic DNA was used for library preparation with the Ligation Sequencing kit (SQK-LSK109) from ONT using the manufacturer's protocols. Base calling of the reads from the three MinION flow cells was performed with guppy v4.0.11 (https://nanoporetech.com/nanopore-sequencing-data-analysis), under default settings. Afterwards, ONT reads quality was checked with Nanoplot v1.28.1 (https://github.com/wdecoster/NanoPlot) and reads shorter than 1,000 bases and mean quality below eight were discarded by running Nanofilt v2.6.0 (https://github.com/wdecoster/nanofilt).

A mix of different tissues (liver, heart, gonads, brain, kidney, muscle) was ground into small pieces using steel balls and a Retsch Mill. A total of 120 mg of the tissue was shipped on dry ice to Novogene (UK) for Illumina paired-end 150 RNA-seq of a 250–300 bp insert cDNA library.

### Genome Size Estimation

Genome size was estimated following a flow cytometry protocol with propidium iodide-stained nuclei described in Hare and Johnston (2012). Ear tissue of one frozen (−80°C) adult sample of *N. procyonoides* and neural tissue of the internal reference standard *Acheta domesticus* (female, 1C = 2 Gb) was mixed and chopped with a razor blade in a petri dish containing 2 ml of ice-cold Galbraith buffer. The suspension was filtered through a 42-μm nylon mesh and stained with the intercalating fluorochrome propidium iodide (PI, Thermo Fisher Scientific) and treated with RNase II A (Sigma-Aldrich), each with a final concentration of 25 μg/ml. The mean red PI fluorescence signal of stained nuclei was quantified using a Beckman-Coulter CytoFLEX flow cytometer with a solid-state laser emitting at 488 nm. Fluorescence intensities of 5,000 nuclei per sample were recorded. We used the software CytExpert 2.3 for histogram analyses. The total quantity of DNA in the sample was calculated as the ratio of the mean red fluorescence signal of the 2C peak of the stained nuclei of the raccoon dog sample divided by the mean fluorescence signal of the 2C peak of the reference standard times the 1C amount of DNA in the standard reference. Six replicates were measured on 6 different days to minimize possible random instrumental errors. Furthermore, we estimated the genome size by coverage from mapping reads used for genome assembly back to the assembly itself using backmap 0.3 (https://github.com/schellt/backmap; Schell et al., 2017). In brief, the method divides the number of mapped nucleotides by the mode of the coverage distribution. By doing so, the length of collapsed regions with many fold increased coverage is taken into account.

### Genome Assembly Workflow

SMRT reads longer than 7 kb were assembled under two different approaches (wtdbg v2.5; Ruan and Li, 2020 and Flye v2.7.1; Kolmogorov et al., 2019). The resulting assemblies were compared in terms of contiguity using Quast v5.0.2 (Gurevich et al., 2013), and evaluated for completeness by BUSCO v3.0.2 (Simão et al., 2015) (under short mode) against the laurasiatheria_odb9 data set (Supplementary Table 1). The assembled genome obtained with Flye presented the highest contiguity and completeness of both approaches and was therefore selected for downstream analyses.

### Scaffolding and Gap Closing

To further improve the assembly, we applied two rounds of scaffolding and gap closing to the selected genome assembly. The genome was first scaffolded with the SMRT reads by SSPACE-longread v1.1 (Boetzer and Pirovano, 2014) and then with ONT reads by SLR (Luo et al., 2019). TGS gapcloser v1.0.1 (Xu et al., 2019) was run after each scaffolding step. Subsequently, Omni-C reads were employed to further scaffold the draft genome following the HiRise pipeline (Putnam et al., 2016) operated by the Dovetail Genomics^TM^ team. The assembly was screened for contamination using BlobTools v1.1.1 (Kumar et al., 2013; Laetsch and Blaxter, 2017) by evaluating coverage, GC content and sequence similarity against the NCBI nt database of each sequence (Figure 1d).

### Transcriptome Assembly

Quality of raw Illumina sequences was checked with FastQC (Andrews, 2010). Low quality bases and adapter sequences were subsequently trimmed by Trimmomatic v0.39 (Bolger et al., 2014) and the transcriptome was assembled using Trinity v2.9.1 (Haas et al., 2013). The transcriptome assembly was evaluated for completeness by BUSCO v3.0.2 against the laurasiatheria_odb9 data set (C: 81.8% [S: 36.0%, D: 45.8%], F:8.0%, M:10.2%). Moreover, the clean RNA-seq reads from different tissues were aligned against the reference genome by HISAT2 (Kim et al., 2015).

### Repeat Annotation

RepeatModeler v2.0 (Smit and Hubley, 2008) was run to construct a *de novo* repetitive library from the assembly. The specific repetitive library was merged with the canid RepBase (Jurka et al., 2005; http://www.girinst.org/repbase/ 18/10/2020), which was further annotated and masked using RepeatMasker v4.1.0 (http://www.repeatmasker.org/).

### Gene Prediction and Functional Annotation

After the repeat sequences were masked, genes were predicted using the homology-based gene prediction tool GeMoMa v1.7.1 (Keilwagen et al., 2016, 2018) and 11 mammalian species as reference organisms. The selected species were *Canis lupus familiaris* (GCF_000002285.3; Lindblad-Toh et al., 2005), *Vulpes vulpes* (GCF_003160815.1; Kukekova et al., 2018), *Mustela erminea* (GCF_009829155.1)*, Zalophus californianus* (GCF_009762305.2), *Ailuropoda melanoleuca* (GCF_002007445.1; Li et al., 2010), *Ursus maritimus* (GCF_000687225.1; Liu et al., 2014), *Felis catus* (GCF_000181335.3; Pontius et al., 2007), *Sus scrofa* (GCF_000003025.6; Fang et al., 2012), *Bos taurus* (GCF_002263795.1; Zimin et al., 2009), *Mus musculus* (GCF_000001635.26; Church et al., 2009), and *Homo sapiens* (GCF_000001405.39; Craig Venter et al., 2001). First, from the mapped RNA-seq reads, introns were extracted and filtered by the GeMoMa modules ERE and DenoiseIntrons. Then, we independently ran the module GeMoMa pipeline for each reference species using MMseqs2 (Steinegger and Söding, 2017) as alignment tools and including the mapped RNA-seq data. Finally, the 11 gene annotations were combined into a final annotation by using the GeMoMa modules GAF and AnnotationFinalizer.

Predicted genes were annotated by BLAST search against the Swiss-Prot database with an e-value cutoff of 10^−6^. InterProScan v5.39.77 (Quevillon et al., 2005) was used to predict motifs and domains, as well as Gene ontology (GO) terms.

### Code Availability

The execution of this work involved using many software tools, for which settings and parameters are described below. Software tools indicated within brackets are dependencies employed during the execution of the main indicated tools. All the tools employed in this work are listed in Supplementary Table 3.

#### Genome Assembly

**(1) Flye v2.7.1:** parameters: –genome-size 3.198g –asm-coverage 40; **(2) sspace-longread v1.1** [bedtools v2.28.0]: all parameters were set as default; **(3) TGS-gapcloser v1.0.1** [minimap2 v2.17 racon v1.4.3]: parameters: –tgstype pb; **(4) SLR** [bwa v0.7.17 samtools v1.10]: parameters: **4.1**: bwa index, **4.2** bwa mem, **4.3** samtools view -Sb, **4.4** bwa mem -k11 -W20 -r10 -A1 -B1 -O1 -E1 -L0 -a -Y, **4.5** samtools view -Sb, **4.6** SLR all parameters were set as default; **(5) TGS-gapcloser v1.0.1** [minimap2 v2.17 racon v1.4.3]: parameters: –tgstype ont; **(6) HiRise pipeline**: all parameters were set by Dovetail Genomics team; **(7) BUSCO v3.0.2** [python v3.7.4 augustus v3.3.2]: parameters: -l /laurasiatheria_odb9/ -m geno; **(8) Blobtools v1.1.1** [samtools v1.10 ncbi-blast v2.10.0]: parameters: **8.1** samtools index, **8.2** blobtools map2cov, **8.3** blastn -task megablast -outfmt '6 qseqid staxids bitscore std' -max_target_seqs 1 -max_hsps 1 -evalue 1e-25, **8.4** blobtools create, view and plot all parameters were set as default; (9) **Jupiterplot v1.0** [minimap2 v2.17 samtools v1.10 circos v0.69-9]: parameters: ng=99 t=64 m=2860953 g=1.

#### Genome Annotation

**(1) RepeatModeler v2.0**: parameters: -pa 16 -LTRStruct; **(2) RepeatMasker v4.1.0**: parameters: -s -pa 18 -no_is -xsmall; **(3) hisat v2.1.0**: parameters: -k 3 –pen-noncansplice 12 -S; **(4) GeMoMa v.1.7.1** [java v1.8.0_221]: **4.1** java -Xmx30G -jar GeMoMa-1.7.1.jar CLI ERE c=TRUE; **4.2** java -Xmx30G -jar GeMoMa-1.7.1.jar CLI DenoiseIntrons coverage_unstranded; **4.3** java -Xmx30G -jar GeMoMa-1.7.1.jar CLI GeMoMaPipeline tblastn=false r=EXTRACTED introns coverage_unstranded DenoiseIntrons.m=100000 GeMoMa.m=100000 GeMoMa.Score=ReAlign AnnotationFinalizer.r=NO o=true; **4.4** java -Xmx30G -jar GeMoMa-1.7.1.jar CLI GAF; **4.5** java -Xmx30G -jar GeMoMa-1.7.1.jar CLI AnnotationFinalizer u=YES i c=UNSTRANDED coverage_unstranded; **4.6** java -Xmx30G -jar GeMoMa-1.7.1.jar CLI Extractor p=true c=true; **(5) BUSCO v3.0.2** [python v3.7.4 augustus v3.3.2]: parameters: -l /laurasiatheria_odb9/ -m prot; **(6) Interproscan v5.39.77**: parameters: -f tsv -iprlookup -pa -goterms -exclappl SignalP_GRAM_NEGATIVE,SignalP_GRAM_POSITIVE -dp; **(7) ncbi-blast v.2.10.0**: parameters: **7.1** makeblastdb -in uniprot_sprot_2020_04.fasta -parse_seqids -dbtype prot, **7.2** blastp -evalue 1e-6 -max_hsps 1 -max_target_seqs 1 -outfmt 6.

#### Genome Size Estimation

**(1) backmap.pl v0.3** [minimap2 v2.17, samtools v1.10, qualimap v2.2.1, bedtools 2.28.0, Rscript v3.6.3, multiqc 1.9]: parameters: -pb -v.

## Data Validation

### Genome Size Validation

The calculated DNA content through flow cytometry experiments was 3.10 Gb, similar to previous flow cytometry studies (3.19 Gb; Wurster-Hill et al., 1988). The genome size estimation by read coverage resulted in 3.23 Gb. Although our draft genome assembly was smaller than the values obtained by flow cytometry and coverage, the assembly length obtained of 2.39 Gb was in the range of other Carnivora genomes (Table 1, Supplementary Table 2) and showed good completeness with 92.9% completely recovered BUSCOs. The difference regarding assembly vs. estimated genome size could be explained by the complex chromosome structure of the raccoon dog which presents large chromatin proximal regions and a fluctuating number of B chromosomes (Duke Becker et al., 2011; Makunin et al., 2018). Both uncommon structures in carnivores are mostly compound by repetitive elements that were most likely not properly resolved and collapsed.

**Table 1:** **A**. Genome assembly and annotation statistics for raccoon dog (*Nyctereutes procyonoides*) and comparison with related species. **B**. Repeat statistics: *De novo* and homology based repeat annotations as reported by RepeatMasker and RepeatModeler; Families of repeats included here are long interspersed nuclear elements (LINEs), short interspersed nuclear elements (SINEs), long tandem repeats (LTR), DNA repeats (DNA), unclassified (unknown) repeat families, small RNA repeats (SmRNA), and others (consisting of small, but classified repeat groups). The total is the total percentage of base pairs made up of repeats in each genome assembly, respectively. **C**. Number and percentage of functional annotated predicted protein-coding genes.

	***Nyctereutes procyonoides***	***Vulpes vulpes***	***Canis lupus familiaris***
**A. GENOME STATISTICS**			
Total sequence length	2,387,080,371	2,406,519,287	2,410,976,875
No. of contigs	877	122,687	27,144
contigs >50,000 bp	233	13,920	10,416
No. of scaffolds	810	24,706	3,268
scaffolds >50,000 bp	179	492	301
Scaffold N50	53,959,811	12,607,163	63,241,923
Scaffold L50	17	54	15
GC content (%)	41.28	41.06	41.31
BUSCO			
Genome			
Complete	92.9% (S:91.5%; D: 1.4%)	92.3% (S:91.1%; D: 1.2%)	93.0% (S:91.6%; D: 1.4%)
Fragmented	3.9%	4.1%	3.6%
Missing	3.2%	3.6%	3.4%
Annotation			
Complete	98.9%(S:38%; D:60.9%)	97.9% (S:57.4%; D:40.5%)	98.2%(S:41.7%; D: 56.5%)
Fragmented	0.8%	1.7%	1.4%
Missing	0.3%	0.4%	0.4%
**B. REPEATS**			
LINE	1,631,835	1,398,679	857,579
SINE	1,252,244	1,651,461	1,503,465
LTR	392,967	423,087	302,932
DNA	294,798	388,850	321,141
Unclassified	15,049	5,253	14,466
SmRNA	1,002,088	44,426	1,110,467
Others	954,227	1,007,275	1,038,344
Total (%)	34.04	39.67	42.13
**C. FUNCTIONAL ANNOTATION**			
InterproScan	78,944 (99.41%)	37,861 (99.89%)	56,849 (99.60%)
GO	61,756 (77.77%)	29,799 (78.62%)	44,539 (78.03%)
Reactome	31,180 (39.26%)	14,809 (39.07%)	21,160 (37.07%)
SwissProt	77,152 (97.16%)	37,616 (99.25%)	56,818 (99.54%)

### Comparison With Other Carnivora Genomes

A total of ~293 Gb raw data, representing 94.5X coverage, was generated using PacBio Sequel II and employed for genome assembly with Flye. After scaffolding with long reads and Omni-C data, we produced a draft genome assembly of 2.39 Gb with a scaffold N50 of 54 Mb (Table 1, section A). The final assembly of the raccoon dog draft genome contained 810 scaffolds (plus mitochondrion), where the largest scaffold was 121,018,622 bp in length which corresponded to the X-sex chromosome. We predicted 27,177 genes in the *N. procyonoides* genome by using a homology-based gene prediction. Among the identified proteins, 61,756 (77.8%) were annotated to have at least one GO term. Finally, 78,944 proteins (99.4%) were assigned to at least one of the databases from InterProScan (Table 1, section C). BUSCO and functional annotation results indicated high quality (Table 1, Supplementary Table 2). We also compared synteny between raccoon dog and dog genome assemblies by running Jupiterplot v1.0 (https://github.com/JustinChu/JupiterPlot). The Jupiterplot displays the largest 58 raccoon dog scaffolds, which covered more than 99% of the dog genome (Figure 1e). The colored bands represent synteny between both genome assemblies. The plot shows high synteny between both genomes with several genomic rearrangements and break points, some of them previously identified (Duke Becker et al., 2011). All these results makes the *N. procyonoides* genome the best genome recovered so far for the Vulpini tribe.

### SARS-CoV-2

Animal cell infection by SARS-CoV-2 is determined by specificity between the receptor-binding domain (RBD) spike protein (S-protein) of SARS-CoV-2 and the membrane proteins ACE2 (peptidase domain of angiostensin I converting enzyme 2) and TMPRSS2 (transmembrane serine protease) (Lam et al., 2020). We identified both proteins in the raccoon dog genome annotation, showing high similarity with dog and fox orthologues. ACE2 protein alignments between dog and raccoon dog showed 99.3% of similarity, with only 6 of 894 different amino acids (Supplementary Figure 1). Moreover, the affinity in the binding process between S-protein from SARS-CoV-2 and ACE2 have been found to be smaller for groups like canids (*Canis, Vulpes*), chiroptera (*Rhinophus, Pteropus*) and pangolins (*Manis*) among others due to the matching of 14 of the 20 key amino acids in human ACE2 protein (Luan et al., 2020a). However, the reported infections of SARS-CoV-2 in domestic dogs and ferrets (Elbe and Buckland-Merrett, 2017; Shu and McCauley, 2017; Shi et al., 2020) indicated that the raccoon dog can be considered as a potential host and vector for this virus along its natural distribution range in East Asia and also in its introduced populations within Europe.

## Data Availability Statement

All raw data generated for this study (PacBio, MinION, Omni-C and RNA-seq reads) are available at the European Nucleotide Archive database (ENA) under the Project number: PRJEB41734, https://www.ebi.ac.uk/ena, PRJEB41734. The final genome assembly and annotation can be found under the accession number GCA_905146905, https://www.ebi.ac.uk/ena, GCA_905146905.

## Ethics Statement

Ethical review and approval was not required for the animal study because the animal was culled in full accordance to German hunting laws (waidgerecht), which means that unnecessary suffering was avoided. Moreover, the individual was not killed for the study. We used one that was killed anyway in accordance to the Convention on Biological Diversity CBD (in § 8h), that stipulates precaution, control and eradication of invasive species as a goal and task of nature conservation under international law. In 2000, the states committed themselves to developing national strategies in Decision V/8(6).

## Author Contributions

SK, JK, and MP conceived this study. JK and CG prepared the samples. CG conducted lab work. LC performed bioinformatic analyses and data statistics with support of TS. LC, JK, AJ, TS, and MP discussed and interpreted the data. LC wrote the manuscript and all authors commented and revised the manuscript.

## Conflict of Interest

The authors declare that the research was conducted in the absence of any commercial or financial relationships that could be construed as a potential conflict of interest.
