# Supplementary material for: De novo Genome Assembly of the Raccoon Dog (Nyctereutes procyonoides)
Source: Front Genet. 2021 Apr 29;12:658256. doi: 10.3389/fgene.2021.658256 (PMC8117329; doi:10.3389/fgene.2021.658256)
Supplement: Supplementary file 1 [file Data_Sheet_1.docx]

***De novo* genome assembly of the raccoon dog (*Nyctereutes procyonoides*)**

Luis J. Chueca^1,2^, Judith Kochmann^1,3^, Tilman Schell^1^, Carola Greve^1^, Axel Janke^1,3,4^ Markus Pfenninger^1,3,5^ and Sven Klimpel^1,3,4^

^1^ LOEWE-Centre for Translational Biodiversity Genomics (LOEWE-TBG), Senckenberg Nature Research Society, Frankfurt am Main, Germany.

^2^ Department of Zoology and Animal Cell Biology, University of the Basque Country (UPV-EHU), Vitoria-Gasteiz, Spain.

^3^ Senckenberg Biodiversity and Climate Research Centre (SBiK-F), Frankfurt am Main, Germany.

^4^ Institute for Ecology, Evolution and Diversity, Goethe University, Frankfurt am Main, Germany.

^5^ Institute of Organismic and Molecular Evolution (iOME), Faculty of Biology, Johannes Gutenberg University, Mainz, Germany.

corresponding author: luisjavier.chueca@ehu.eus

**Supplementary Material**

**Extended Material and Methods.**

*SMRTbell library*

Total input DNA was approximately 600 ng. Ligation with T-overhang SMRTbell adapters was performed at 20°C overnight. Following ligation, the SMRTbell library was purified with an AMPure PB bead clean up step with 0.45X volume of AMPure PB beads. Subsequently a size-selection step with AMPure PB Beads was performed to remove short SMRTbell templates < 3kb. For this purpose the AMPure PB beads stock solution was diluted with elution buffer (40% volume/volume) and then added to the DNA sample with 2.2X volume.

Sequencing primer v4 and Sequel® II Polymerase 2.0 were annealed and bound, respectively, to the SMRTbell library. The library was loaded at an on-plate concentration of 60 pM using diffusion loading. CLR mode on the Sequel System II was run for 30 hour movie time with NO pre-extension and Software SMRTLINK 8.0.

*Omni-C library*

The Omni-C™ library preparation started with endogenous chromatin being fixed in place (cross-linked) to preserve the genome conformation. The crosslinked DNA was then digested using a sequence independent endonuclease. Free ends were ligated in a two-step process that incorporates a biotinylated bridge between chromatin ends preserving both short- and long-range DNA contiguity. The DNA was then purified and sheared to a size appropriate for Illumina short-read sequencing. After shearing, the biotinylated fragments were enriched to assure that only fragments originating from ligation events are sequenced in paired-end mode on an Illumina platform. The library preparation was done in 3 steps (3 days):

Day 1: Stage 1 & 2 (crosslink / nuclease digestion and quantification)

- Tissue was grinded in with mortar and pestle in LN_2_
- Started with Appendix 2: sample preparation (Stage 1) and Lysate Quantification (Stage 2) including Nuclease Enzyme Titration
- Centrifugation was done for 5min at 3.000 x g (instead of 2.000 x g)
- Lysate quantification was with SPRIselect beads from Beckmann & Coulter instead of Zymo Genomic DNA Clean & Concentrator
- CDE / CDI values for QC-A and QC-C was in range, therefore both samples were used for Stage 3.

Day 2: Stage 3: (end polishing / bridge ligation / intra-aggregate ligation and crosslink reversal)

- All steps were done according to protocol (version 1.1)
- Needed DNA amount is 1,000 ng, we had 52 ng in total (library A) and 25 ng (library C)

Day 3: stage 4 & 5: (end repair / adapter ligation / ligation capture and index PCR)

- Needed DNA amount is 150 ng in total, we had 45 ng in total (library A) and 5 ng in total (library C)

Index PCR: because of the low amount of DNA we decided to increase the number of cycles from 12 to 18 cycles.

**Supplementary Tables and Figures:**

**Table S1**: Comparison between draft genomes assemblies obtained by the different tools.

|  | **wtdbg v2.5** | **Flye v2.7.1** |
| --- | --- | --- |
| **Number of contigs** | 3,922 | 1,165 |
| **Largest contig** | 82,483,431 | 75,247,497 |
| **Contig N50** | 24,814,655 | 33,220,459 |
| **Total length** | 2,343,115,400 | 2,343,874,447 |
| **BUSCO n=6253**  **Complete [Single-copy (S), Duplicated (D)]**  **Fragmented**  **Missing** | 86.7% (S:85.5%; D: 1.2%)  6.4%  6.9% | 92.9% (S:91.5%; D: 1.4%)  3.9%  3.2% |

**Table S2. Genome comparison between raccoon dog (*Nyctereutes procyonoides*) and other Carnivora species.**

|  |  | **Carnivora** | | | | | | | |
| --- | --- | --- | --- | --- | --- | --- | --- | --- | --- |
|  |  | Canidae | | | Mustelidae | Otariidae | Ursidae | | Felidae |
|  |  | *Nyctereutes procyonoides* | *Vulpes vulpes* | *Canis lupus familiaris* | *Mustela erminea* | *Zalophus californianus* | *Ursus maritimus* | *Ailuropoda melanoleuca* | *Felis silvestris catus* |
| **Number** |  |  |  |  |  |  |  |  |  |
|  | **Gene** | 27,177 | 21,427 | 29,253 | 27,875 | 24,899 | 23,470 | 27,172 | 28,653 |
|  | **mRNA** | 79,410 | 37,902 | 57,079 | 60,430 | 61,011 | 28,874 | 55,247 | 54,335 |
|  | **CDS** | 922,301 | 449,276 | 708,818 | 770,332 | 740,920 | 286,832 | 638,826 | 675,451 |
| **Mean** |  |  |  |  |  |  |  |  |  |
|  | **mRNAs/gene** | 2.92 | 1.77 | 1.95 | 2.17 | 2.45 | 1.23 | 2.03 | 1.9 |
|  | **CDSs/mRNA** | 11.61 | 11.85 | 12.42 | 12.75 | 12.14 | 9.93 | 11.56 | 12.43 |
| **Median length** |  |  |  |  |  |  |  |  |  |
|  | **Gene** | 11,574 | 16,322 | 13,980 | 14,264 | 14,965 | 13,907 | 12,362 | 13,181 |
|  | **mRNA** | 23,821 | 25,937 | 32,462 | 30,597 | 30,721 | 20,247 | 26,804 | 31,448 |
|  | **CDS** | 123 | 123 | 121 | 123 | 123 | 121 | 124 | 122 |
| **Total space (Mb)** |  |  |  |  |  |  |  |  |  |
|  | **Gene** | 880.39 | 969.67 | 1,159.48 | 1,201.48 | 1,144.88 | 920,01 | 1,114.21 | 1,230.61 |
|  | **mRNA** | 880,39 | 930.52 | 1,008.77 | 1,088.63 | 1,079.036 | 841.77 | 1,031.15 | 1,095.64 |
|  | **CDS** | 40.12 | 33.38 | 33.59 | 36.32 | 35.76 | 32.07 | 35.97 | 34.76 |
| **Single** |  |  |  |  |  |  |  |  |  |
|  | **CDS mRNA** | 4,084 | 2,793 | 3,682 | 4,536 | 5,114 | 2,474 | 5,307 | 3,427 |
| **BUSCO**  **n:6253** | **Complete** | 98.90%  (S: 38.00%; D:60.90%) | 97.90%  (S: 57.40%; D: 40.50%) | 98.20%  (S: 41.70%; D: 56.50%) | 99.20%  (S: 39.30%; D: 59.30%) | 99.40%  (S: 36.30%; 63.10%) | 97.10%  (S: 71.80%; D: 25.30%) | 98.10%  (S: 44.30%; D:53.80%) | 99.20% (S:42.60%; D:56.60%) |
|  | **Fragmented** | 0.80% | 1.70% | 1.40% | 0.30% | 0.50% | 2.60% | 1.60% | 0.40% |
|  | **Missing** | 0.30% | 0.40% | 0.40% | 0.50% | 0.10% | 0.30% | 0.30% | 0.40% |

**Table S3**. Software employed in this work, their package version and source availability.

| **Name** | **Version** | **Url** |
| --- | --- | --- |
| Flye | 2.7.1 | https://github.com/fenderglass/Flye |
| wtdbg2 | 2.5 | https://github.com/ruanjue/wtdbg2 |
| Quast | 5.0.2 | https://github.com/ablab/quast |
| BUSCO | 3.0.2 | https://busco.ezlab.org/ |
| Blobtools | 1.1.1 | https://github.com/DRL/blobtools |
| SSPACE-longread | 1.1 |  |
| SLR |  | https://github.com/luojunwei/SLR |
| TGS gapcloser | 1.0.1 | https://github.com/BGI-Qingdao/TGS-GapCloser |
| FastQC | 0.11.9 | https://www.bioinformatics.babraham.ac.uk/projects/fastqc/ |
| Trimmomatic | 0.39 | http://www.usadellab.org/cms/?page=trimmomatic |
| MultiQC | 1.9 | https://multiqc.info/ |
| Trinity | 2.9.1 | https://github.com/trinityrnaseq/trinityrnaseq/wiki |
| GeMoMa | 1.6.4 | http://www.jstacs.de/index.php/GeMoMa |
| MMseqs2 |  | https://github.com/soedinglab/MMseqs2 |
| guppy | 4.0.11 | https://nanoporetech.com/nanopore-sequencing-data-analysis |
| Nanoplot | 1.28.1 | https://github.com/wdecoster/NanoPlot |
| Nanofilt | 2.6.0 | https://github.com/wdecoster/nanofilt |
| backmap.pl | 0.3 | https://github.com/schellt/backmap |
| SAMtools | 1.10 | https://github.com/samtools/samtools |
| BWA | 0.7.17 | https://github.com/lh3/bwa |
| minimap2 | 2.17 | https://github.com/lh3/minimap2 |
| Qualimap | 2.2.1 | http://qualimap.conesalab.org/ |
| bedtools | 2.28.0 | https://bedtools.readthedocs.io/en/latest/ |
| Rscript | 3.6.3 | https://www.r-project.org/ |
| RepeatModeler | 2.0 | http://www.repeatmasker.org/RepeatModeler/ |
| RepeatMasker | 4.1.0 | http://www.repeatmasker.org/ |
| JupiterPlot | 1.0 | https://github.com/JustinChu/JupiterPlot |
| circos | 0.69-9 | http://circos.ca/software/download/circos/ |
| HISAT2 | 2.1.0 | http://daehwankimlab.github.io/hisat2/ |


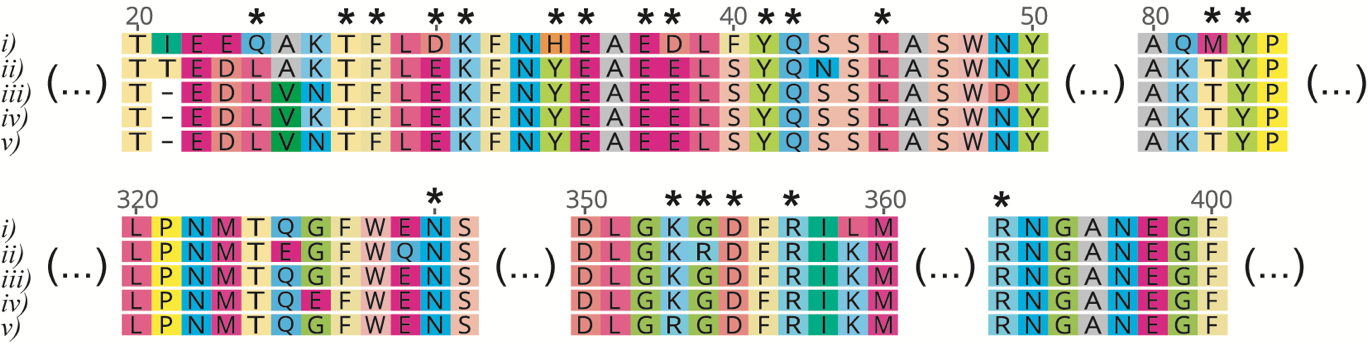


**Figure S1.** ACE2 protein orthologues alignment between human (*i*), stoat (*ii*), red fox (*iii*), dog (*iv*) and raccoon dog (*v*). The 20 key amino acids (Luan et al., 2020a) in the binding process between spike-protein of SARS-CoV-2 and ACE2 are indicated by asterisks.
